# Comparison of Oral Procainamide and Mexiletine Treatment of Recurrent and Refractory Ventricular Tachyarrhythmias

**DOI:** 10.3390/jcm13206099

**Published:** 2024-10-13

**Authors:** Mauro Toniolo, Daniele Muser, Giacomo Mugnai, Luca Rebellato, Elisabetta Daleffe, Claudio Bilato, Massimo Imazio

**Affiliations:** 1 Division of Cardiology, University Hospital “S.Maria della Misericordia”, P.le S.Maria della Misericordia 15, 33100 Udine, Italy; 2 Division of Cardiology, West Vicenza General Hospital, 36071 Arzignano, Italy

**Keywords:** ventricular arrhythmias, antiarrhythmic therapies, arrhythmic burden, implantable cardioverter defibrillators

## Abstract

**Background:** Antiarrhythmic therapy for recurrent ventricular arrhythmias (VAs) in patients having undergone catheter ablation and in whom amiodarone and/or beta-blockers were ineffective or contraindicated is a controversial issue. **Purpose:** The present study sought to compare the efficacy and tolerability of oral procainamide and mexiletine in patients with recurrent ventricular arrhythmias when the standard therapy strategy failed. **Methods:** All patients with an implantable cardioverter defibrillator (ICD) treated with oral procainamide or mexiletine for recurrent ventricular tachycardia (VT) or ventricular fibrillation (VF) in two cardiology divisions between January 2010 and January 2020 were enrolled. Patients were divided into group A (oral procainamide) and group B (mexiletine) and the two groups were compared to each other. The primary endpoint was the efficacy of therapy; the secondary endpoint was the discontinuation of therapy. All events that occurred during procainamide or mexiletine treatment were compared with a matched duration period before the initiation of the therapy. Antiarrhythmic therapy was considered effective when a ≥80% reduction of the sustained ventricular arrhythmias burden recorded by the ICD was achieved. **Results:** A total of 68 consecutive patients (61 males, 89.7%; mean age 74 ± 10 years) were included in this retrospective analysis. After a median follow-up of 19 months, 38 (56%) patients had a significant reduction in the VA burden. After multivariable adjustment, therapy with procainamide was independently associated with an almost 3-fold higher efficacy on VA suppression compared to mexiletine (HR 2.54, 95% CI 1.06–6.14, *p* = 0.03). Only three patients (9%) treated with procainamide presented severe side effects (dyspnea or hypotension) requiring discontinuation of therapy compared with six patients (18%) treated with mexiletine who interrupted therapy because of severe side effects (*p* = 0.47). **Conclusions:** Compared to mexiletine, oral procainamide had a higher efficacy for the treatment of recurrent and refractory VAs, and showed a good profile of tolerability.

## 1. Introduction

**Figure 1:**
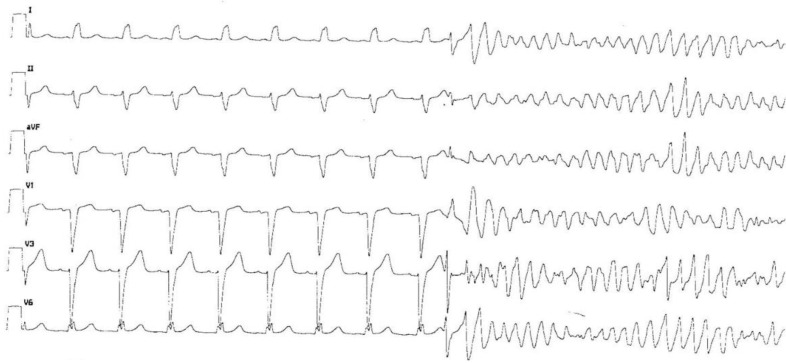
Ventricular fibrillation induced by a ventricular extrasystole in a patient with a dilated cardiomyopathy with left bundle branch block.

Refractory ventricular tachycardia (VT) in patients with structural heart disease (Figure 1) and an implantable cardioverter defibrillator (ICD) may induce repeated shocks, which degrades living conditions, exacerbate a setting of heart failure, and finally, cause an increase in the mortality rate [1,2,3,4,5]. Currently, catheter ablation of VT is the favorite therapy after repeated ICD interventions due to its higher effectiveness and safety over antiarrhythmic drugs (AADs) [6]. When amiodarone and/or beta-blockers are ineffective or contraindicated in patients already submitted to previous catheter ablations, their clinicians face a controversial problem. In this situation, there are treatment strategies not supported by concrete evidence, including other AADs, unconventional ablation techniques (bipolar or alcohol ablation), and renal or cardiac sympathetic denervation.

Mexiletine is a sodium channel blocker, the oral lidocaine equivalent, and a class IB AAD. Mexiletine is used in both cardiology as an AAD and in neurology for the treatment of myotonic dystrophy. Initially, mexiletine was tested as an AAD, and the first results were presented in 1973 [7]. Mexiletine has a half-life of 10 to 12 h and is metabolized by the liver in small quantities. This drug can be given three times daily; therefore, it can be eligible as a chronic therapy. The recommended dosing schedule is 100–300 mg every 6 or 8 h. In the long run, other AADs, such as sotalol and amiodarone, overtook mexiletine for reasons relating to efficacy and safety [8]. However, it can still be an effective drug in adult patients with refractory VT or ventricular fibrillation (VF) when other therapies are not able to control the arrhythmias, and in adult and pediatric patients with long QT syndrome. The use of mexiletine has also been recommended in international guidelines for the management of VT/VF [9].

Subsequent to the use of mexiletine, various side effects, mostly connected to the gastrointestinal tract and nervous system, may occur. The main side effects consist of nausea, sickness, vertigo, tremor, tenseness, headache, and liver function abnormalities. Procainamide is a class IA antiarrhythmic that binds to fast sodium channels inhibiting recovery after repolarization. Procainamide and its metabolite *N*-acetyl procainamide also have potassium efflux channel blocking effects, prolonging the QT interval. The half-life of procainamide is 2.5 to 5 h. Long-term oral therapy typically uses dosages in the range of 15 to 50 mg/kg/day, fractionated every 4 to 6 h. Procainamide is metabolized hepatically. The majority of procainamide-related side effects are based on the plasma concentration and the length of therapy. The most frequent side effect is a systemic lupus erythematosus-like syndrome. Manifestations can include fevers, arthralgias, myalgias, rashes, and, less often, kidney and liver toxicities. In the 1950s, it was demonstrated to diminish or abolish ventricular arrhythmias (VAs) in acute settings in up to 90% of patients with premature ventricular contractions (PVCs) and 80% of patients with VT [10,11,12]. In the previous guidelines, oral procainamide was included in a list of available drugs, but no specific recommendations were given about its use [13].

Purpose: The present study sought to compare the efficacy and tolerability of oral procainamide and mexiletine in patients with an ICD suffering heart failure and recurrent VAs when the standard therapy strategy failed.

## 2. Methods

All patients with an ICD who were treated with oral procainamide or mexiletine for recurrent VT/VF in two cardiology divisions from January 2010 to January 2020 were included in this study. Implantable cardioverter defibrillators were implanted according to the guidelines in effect at the time of implantation as either primary prevention for patients with dilated cardiomyopathy and a left ventricular ejection fraction ≤ 35%, or secondary prevention for those with prior VT/VF without reversible causes [13]. There were no restrictions regarding age, type of cardiomyopathy, accompanying diseases, or prior interventions, and no exclusion criteria were applied. Given the short half-life of both procainamide and mexiletine in tablet form, each drug was administered three times a day (every 8 h).

Participants were divided into two groups: group A (oral procainamide) and group B (mexiletine), with the two groups compared to one another. The primary endpoint was the efficacy of therapy, while the secondary endpoint was the ending of therapy. All events that occurred during treatment with procainamide or mexiletine were compared to a matched control period prior to starting the therapy. Antiarrhythmic therapy was deemed effective if there was a ≥80% scaling down of the VAs burden registered by the ICD. An electrocardiogram (ECG) was regularly performed 10 days after initiating pharmacological treatment, and these results were compared to a baseline ECG. Echocardiograms were conducted both before treatment began and after treatment cessation, or at the last follow-up if the drug was still being administered. Antitachycardia pacing protocols and detection rates for ICD shocks were tailored to each individual rather than uniformly programmed.

### 2.1. Statistical Analysis

Continuous variables were presented as the mean ± standard deviation (SD) or as the median with an interquartile range, and comparisons were made using either analysis of variance (ANOVA) or the Kruskal–Wallis H-test, as appropriate. Categorical variables were presented as counts and percentages, with comparisons conducted using the chi-squared test or Fisher’s exact test, as appropriate. The comparison of ventricular arrhythmias before and after treatment with procainamide and mexiletine was performed using the Wilcoxon’s test due to the non-Gaussian distribution of data. Statistical analyses were performed using SPSS version 25.0 (IBM SPSS Inc., Chicago, IL, USA), with a two-tailed *p*-value of <0.05 deemed statistically significant.

### 2.2. Ethical Aspects

This was a retrospective study involving humans. Informed consent was waived owing to the use of retrospective and de-identified data. The research reported in this paper adhered to the CONSORT guidelines for the reporting of clinical trials and the guidelines set forth by the Helsinki Declaration. This study was approved by the institutional review committee of University Hospital S.Maria della Misericordia of Udine (Italy) with the code STUD511110 on 9 April 2022.

This research was presented as an abstract at the 2022 Congress of the European Heart Rhythm Association, and an extract of that abstract was reported in a dedicated supplement of *Europace* (Vol. 24, Issue Supplement 1, May 2022). The copyright belongs to the authors.

## 3. Results

A total of 68 consecutive patients (61 males, 89.7%; mean age 74 ± 10 years) were included in this retrospective analysis. Baseline clinical characteristics are reported in Table 1. Most patients had dilatative cardiomyopathy (88%), primarily of ischemic origin (65%). The average left ventricular ejection fraction was 34 ± 11%, with most patients as New York Heart Association Class II (71%) and having a dual-chamber ICD (56%). Cardiac resynchronization therapy was used in only 29% of patients. Importantly, 77% of patients received an ICD for secondary prevention, identifying them as a high arrhythmic risk group.

Catheter ablation of VT was attempted in 27 patients (40%) prior to initiating drug therapy. Concurrent treatment with amiodarone was present in 32 (47%) patients, with oral procainamide or mexiletine added when amiodarone alone was ineffective in controlling VAs. Overall, amiodarone therapy was attempted in 91% (62/68) of patients, but was discontinued in 26 patients due to side effects, including drug-induced hyperthyroidism in 14 patients, pulmonary fibrosis in 5, photosensitivity in 1, QT interval prolongation in 1, hypothyroidism in 1, and general malaise with symptoms like nausea, sialorrhea, or cold sweats in 4 patients.

**Figure 2:**
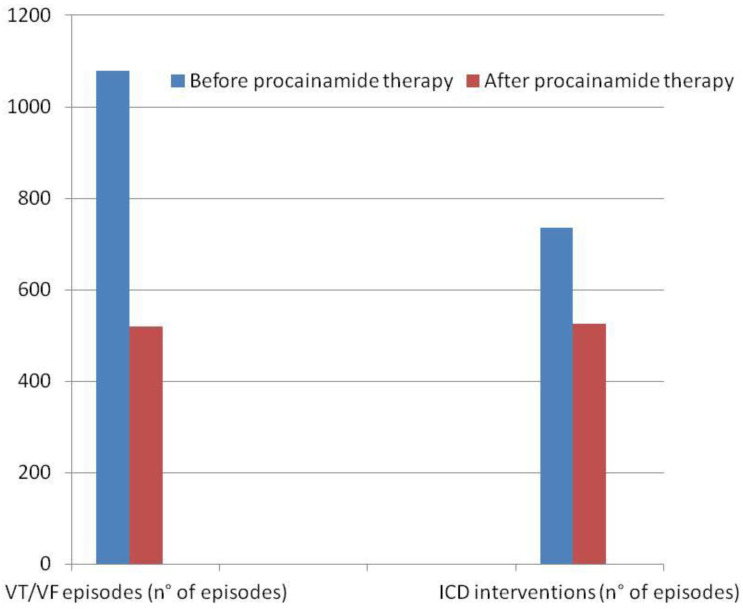
Histogram showing the number of VT/VF episodes and the number of appropriate implantable cardioverter defibrillator ICD interventions before and after oral procainamide administration.

**Figure 3:**
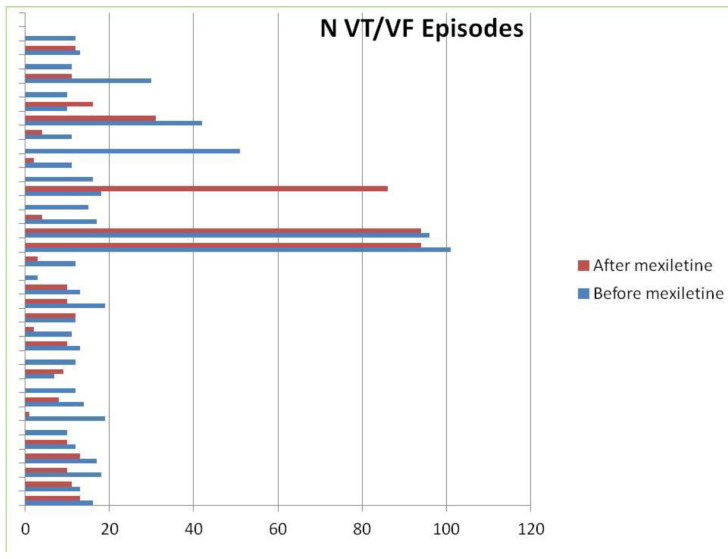
Plot showing the frequency (N) of VT/VF after (red lines) and before (blue lines) mexiletine administration. Each line represents an individual patient. Note that in a few patients there are no red lines matching blue lines (no VT/VF after mexiletine administration).

None of the patients included in our study received flecainide, propafenone, quinidine, nadolol, or propanolol, and only two patients underwent treatment with sotalol. In the procainamide group, 62% (21/34) were on concurrent amiodarone, compared with 32% (11/34) in the mexiletine group. The initial dose of procainamide was 900 mg per day, adjusted according to clinical response, with a mean dose of 1207 ± 487 mg/day. Mexiletine was started at 600 mg/day, with a mean dose of 576 ± 66 mg/day. After a median follow-up of 19 months, 38 patients (56%) showed a significant reduction in VA burden. For those treated with procainamide, VA events decreased from 1079 before treatment to 520 after, and ICD therapies were reduced from 736 to 525 (Figure 2). In the mexiletine group, VA events decreased from 697 to 476, and ICD therapies dropped from 349 to 266 (Figure 3). Table 2 and Table 3 present the effects of both therapies on arrhythmic burden and ICD interventions for each group.

In group A, 20 patients had atrial fibrillation prior to treatment. However, after receiving procainamide, stable sinus rhythm was achieved in 23 patients, indicating an improvement in 9 patients. After multivariable adjustment (Table 4), procainamide therapy was independently associated with nearly a 3-fold greater efficacy in VA suppression compared to mexiletine (HR 2.54, 95% CI 1.06–6.14, *p* = 0.03). Similar results were found after adjustment using inverse probability weighting based on a propensity score accounting for age, gender, left ventricular ejection fraction, ischemic heart disease, and concurrent amiodarone therapy (HR 2.89, 95% CI 1.26–6.62, *p* = 0.01). No analysis of the efficacy of procainamide or mexiletine according to the underlying etiology of cardiac disease was conducted.

Severe side effects, including dyspnea or hypotension, led to the discontinuation of procainamide in three patients (9%) compared to six patients (18%) in the mexiletine group (hypotension) (*p* = 0.47).

## 4. Discussion

The main findings of our study are as follows: (1) Only a small number of patients utilized mexiletine or oral procainamide as AADs. (2) These medications were effective in treating VAs within the study population. (3) Oral procainamide appears to have been more effective than mexiletine for recurrent and refractory ventricular arrhythmias and demonstrated a favorable tolerability profile.

A recent systematic review synthesized the available evidence regarding the effectiveness and safety of mexiletine, a well-established AAD, for patients at risk of recurrent VAs. The authors concluded that mexiletine is both effective and safe. Additionally, another recent study demonstrated that mexiletine effectively suppresses VAs and reduces the risk of recurrence in patients with chronic coronary syndrome who have an ICD [14,15].

To our knowledge, there are only two recent studies examining the use of oral procainamide, which yielded different results. Toniolo et al. [16] reported on the treatment of 34 ICD patients who experienced a significant reduction in ICD therapies and VAs. Conversely, Steinberg et al. [17] published an abstract detailing 23 patients in whom oral procainamide had limited efficacy, particularly in those with severe comorbidities, refractory VT, and prior treatment failures with standard AADs. Interestingly, the mean dose of oral procainamide was higher in Steinberg’s study compared to that of Toniolo.

A recent consensus statement from the European Heart Rhythm Association lists oral procainamide among the drugs available to prevent recurrences of electrical storms, providing a range of expected dosages; however, it does not offer specific recommendations for its use [18]. Although there are older studies comparing mexiletine with oral procainamide, these were conducted in an era before the advent of transcatheter ablation and ICDs, making them less relevant to contemporary patient populations. For example, Campbell et al. [19] conducted a controlled study examining the incidence of VAs after myocardial infarction, comparing procainamide, mexiletine, and a placebo. While the active treatments were statistically significant compared to the placebo, there was no notable difference between the active therapies. Mexiletine was found to be easier to administer and had lower toxicity than oral procainamide. In another study by Nademanee et al. [20], the efficacy of amiodarone, mexiletine, and oral procainamide was compared in 30 patients with frequent premature ventricular contractions (PVCs), revealing that mexiletine was significantly less effective than amiodarone, but had an effect similar to that of procainamide.

In our practice, mexiletine and oral procainamide were used interchangeably based on availability. Since the late 1990s, AADs have become less profitable for the pharmaceutical industry, leading to a decrease in their availability in many countries [21]. Both mexiletine and oral procainamide are currently in short supply in Europe, making it challenging for clinicians to access these medications. In Italy, mexiletine was previously produced exclusively by the military pharmacy and distributed sporadically to hospital pharmacies across the country. Currently, it is manufactured by Teva Pharmaceuticals (Parsippany, NJ, USA) and imported from the U.S. Oral procainamide, on the other hand, has been produced in Spain under the name Byocoril^®^ (Urial Group, Madrid, Spain) or compounded by private pharmacies, with costs often borne by patients. At present, mexiletine is imported from the U.S., while oral procainamide is sourced from Japan. In both cases, clinicians must send a request form to the Italian Medicines Agency to obtain these medications at no cost to patients.

There are reported experiences of combination therapy using mexiletine and oral procainamide. In one study, mexiletine was initiated at a daily dose of 600 mg and increased until adverse effects limited further dosing; however, only 15 out of 20 patients tolerated a dose of 900 mg daily. The most common side effects were nausea, indigestion, dizziness, tremor, nystagmus, and weakness. The addition of oral procainamide led to an increased frequency of gastrointestinal symptoms and weakness. Adverse effects typically arose within 48 h after therapy initiation or during dose escalation. Ultimately, early results were unsatisfactory, leading to the discontinuation of patient enrollment [22].

## 5. Conclusions

Both mexiletine and oral procainamide are effective in treating VAs. However, oral procainamide demonstrates greater efficacy in managing recurrent and refractory VAs and has a favorable tolerability profile. The combination of these two drugs is not recommended.

## Figures and Tables

**Table 1:** Clinical characteristics of patients.

60/68 (88%) 44/68 (65%) 6/68 (9%) 2/68 (3%) 2/68 (3%) 2/68 (3%) 5/68 (7%)	Dilatative cardiomyopathy - CAD - Idiopathic - Hypertrophic - Alcoholic - Myocarditis-related - Valvulopathy-related
8/68 (12%) 4/68 (6%) 2/68 (3%) 2/68 (3%)	Non-dilatative cardiomyopathy - CAD - Idiopathic - Valvulopathy-related
34 ± 11%	LVEF [%] (mean, range)
4/48/14/2	NYHA Class: I/II/III/IV
68/68 (100%) 10/68 (1.5%) 38/68 (56%) 20/68 (29%)	ICD - Single chamber - Dual chamber - Biventricular
16/68 (23.5%) 52/68 (76.5%)	Prophylaxis: - Primary - Secondary
42/68 (62%) 34/68 (50%) 15/68 (22%) 34/68 (50%) 25/68 (37%) 10/68 (15%) 35/68 (51%) 3/35 (8.5%) 14/35 (40%) 8/35 (23%) 10/35 (28.5%)	Comorbidities: - Hypertension - Hyperlipidemia - Diabetes - Atrial fibrillation - Chronic renal insufficiency - COPD - Thyroid disease Spontaneous hyperthyroidism Drug-induced hyperthyroidism Spontaneous hypothyroidism Drug-induced hypothyroidism

Table legend: CAD = coronary arteries disease; LVEF = left ventricular ejection fraction; NYHA = New York Heart Association; ICD = implantable cardioverter defibrillator; COPD = chronic obstructive pneumonia diseases.

**Table 2:** Effects of oral procainamide on the arrhythmic burden and ICD interventions.

95% Confidence Interval	Min; Max	Median	SD	Mean	Variable
[26.71; 32.49]	19.00; 38.00	30.00	5.04	29.6	LVEF
[−14.12; 173.38]	1.00; 392.00	19.00	170.38	79.63	VT/VF before
[−1.02; 60.76]	0.00; 140.00	15.00	53.88	29.87	VT/VF after
[−2.08; 74.34]	0.00; 289.00	14.50	69.44	36.13	ATP before
[−1.48; 43.62]	0.00; 158.00	5.00	39.33	21.07	ATP after
[−0.56; 13.62]	0.00; 55.00	1.00	13.38	6.53	ICD shock before
[−1.22; 14.48]	0.00; 53.00	0.00	14.27	6.63	ICD shock after

Table legend: SD = standard deviation; LVEF = left ventricular ejection fraction; VT = ventricular tachycardia; VF = ventricular fibrillation; ATP = antitachycardia pacing; ICD = implantable cardioverter defibrillator.

**Table 3:** Effects of mexiletine on the arrhythmic burden and ICD interventions.

95% Confidence Interval	Min; Max	Median	SD	Mean	Variable
[29.10; 37.12]	10.94; 65.00	31.00	11.93	33.11	LVEF
[12.01; 52.59]	1.00; 101.00	20.00	62.11	32.30	VT/VF before
[1.62; 58.82]	0.00; 96.00	10.50	86.33	30.22	VT/VF after
[2.31; 63.95]	0.00; 603.00	15.00	95.64	33.13	ATP before
[0.92; 48.26]	0.00; 421.00	4.50	71.46	24.59	ATP after
[0.40; 14.20]	0.00; 133.00	2.5	21.42	7.3	ICD shock before
[0.08; 12.10]	0.00; 104.00	0.00	18.14	6.09	ICD shock after

Table legend: SD = standard deviation; LVEF = left ventricular ejection fraction; VT = ventricular tachycardia; VF = ventricular fibrillation; ATP = antitachycardia pacing; ICD = implantable cardioverter defibrillator.

**Table 4:** Multivariable Cox proportional hazards analysis of baseline covariates in relation to effective VAs suppression.

Multivariable		
*p*	HR (95% CI)	
0.92	0.99 (0.97–1.03)	Left ventricular ejection fraction
0.93	1.05 (0.35–3.13)	Ischemic heart disease
0.99	1.01 (0.44–2.29)	Concomitant amiodarone therapy
0.03	2.54 (1.06–6.14)	Procainamide therapy *

* Compared to mexiletine.

## Data Availability

Data are available on reasonable request.
